# Extraction and identification of bioactive compounds (eicosane and dibutyl phthalate) produced by *Streptomyces* strain KX852460 for the biological control of *Rhizoctonia solani* AG-3 strain KX852461 to control target spot disease in tobacco leaf

**DOI:** 10.1186/s13568-017-0351-z

**Published:** 2017-03-03

**Authors:** Taswar Ahsan, Jianguang Chen, Xiuxiang Zhao, Muhammad Irfan, Yuanhua Wu

**Affiliations:** 1 Department of Plant Pathology, College Plant Protection, Shenyang Agricultural University, Shenyang, People’s Republic of China; 2 Department of Biotechnology, University of Sargodha, Sargodha, Pakistan

**Keywords:** *Streptomyces* strain KX852460, *Rhizoctonia solani* AG-3, Gas chromatography–mass spectrometry, Aromatic compounds, Target spot

## Abstract

*Streptomyces* strain KX852460 having antifungal activity against *Rhizoctonia solani* AG-3 KX852461 that is the causal agent of target spot disease in tobacco leaf. The aim of the study was to determine the antifungal activity of *Streptomyces* strain KX852460 extract against *R. solani* AG-3 and to identify bioactive antifungal compounds produced by strain KX852460. Crude substance was produced by submerged fermentation process from *Streptomyces* strain KX852460. Various solvent was used to extract the culture filtrate. Among all, ethyl acetate extracted supernatant showed great potency against *R. solani* AG-3 KX852461. The active fractions were purified by silica gel column chromatography having 52 mm zone of inhibition against *R. solani* AG-3 KX852461. The purified fractions were identified by gas chromatography–mass spectrometry technique. Twenty-seven compounds were identified and most of the compounds were the derivatives of aromatic compounds. Eicosane (C_20_H_42_) and dibutyl phthalate (C_16_H_22_O_4_) were found antifungal compounds in this study. While morphinan, 7,8-didehydro-4,5-epoxy-17-methyl-3,6-bis[(trimethylsilyl)oxy]-, (5.Alpha. 6.Alpha)—(C_23_H_35_NO_3_Si_2_), cyclononasiloxane, octadecamethyl—(C_18_H_54_O_9_Si_9_) and benzoic acid, 2,5-bis(trimethylsiloxy) (C_16_H_30_O_4_Si_3_) were the major compounds with highest peak number. These results suggested that *Streptomyces* strain KX852460 had good general antifungal activity and might have potential biocontrol antagonist against *R. solani* AG-3 KX852461 to cure the target spot in tobacco leaf.

## Introduction

In the Liaoning province of China in 2006 target spot disease of tobacco was investigated, *Rhizoctonia solani* was the causal agent and caused heavy economic loss regarding to the production and quality of the tobacco (Wu et al. 2012). Anastomosis AG-3 of the *R. solani* is the causal agent of target spot in tobacco (Johnk et al. 1993). To conflict with phytopathogens biocontrol is most potent and environment friendly practice (Castano et al. 2013). Against the *R. solani* biocontrol is better strategy even though environmental conditions affected its efficacy (dos Reis Almeida et al. 2007). *Actinomycetes* are extensively present microorganisms in the environment that have potential to produce bioactive compounds against the phytopathogens (Xue et al. 2013; Zeng et al. 2013). Among the *Actinomycetes*, *Streptomyces* is the largest genus and belongs to the family *Streptomycetaceae* (Kämpfer 2006).

*Streptomyces* have potential to produced curial compounds for antibiotics and agro-antibiotics (Demain 2009). *Streptomycetes* species are the source of 75% of antibiotics (Bhavana et al. 2014). Different solvents and elucidation utilized to extract the secondary metabolites and their structure can be finding by different techniques for example gas chromatography–mass spectrometry (GC–MS), liquid chromatography–mass spectrometry (LC–MS), and nuclear magnetic resonance (NMR) (Tiwari et al. 2015). Volatile and semi volatile compounds with lower molecular mass can be separated by using GC–MS (Snyder et al. 2012). The GC–MS is novel technology for isolation of the compounds that present in the secondary metabolites. Recently antibacterial (Khattab et al. 2016), antifungal compound against *Lycopersici* and *Fusarium oxysporum* (Jalaluldeen et al. 2015), antifungal compounds against *Pyricularia oryzae* (Awla et al. 2016), and broad spectrum pharmaceutical compounds (Narasaiah et al. 2014) were extracted by GC–MS. The main objective of this study was extraction, purification and identification of bioactive antifungal compounds produced from *Streptomyces* strain KX852460 grown under submerged fermentation.

## Materials and methods

### Microorganism

*Streptomyces* strain was isolated from soil and identified by 16S rRNA gene sequence technology and sequence was submitted to Gene bank under accession number of KX852460 and also submitted to Chinese general microbial collection center (CGMCC4.7384). The strain was used for the production of antifungal compounds in submerged fermentation. *Rhizoctonia solani* AG-3 was obtained from naturally infected tobacco leaves in Dandong of China which was also identified by 16S rRNA gene sequence technique and sequence obtained was submitted to the Gene bank and under Accession Number of KX852461 and also submitted to Chinese general microbial collection center (CGMCC3.18223). Other test pathogens obtained from the plant pathology lab of college of plant protection, Shenyang agricultural University China. Fungus pathogens were stored on potato dextrose agar (PDA) at 4 °C.

### Preparation of inoculum

Fermentation was performed in two stages, seed growth and production of active antifungal substance. *Streptomyces* Strain KX852460 was grown on plates of Gause’ s synthetic agar medium at 28 °C for 5 days after spore production used in liquid fermentation medium. Two spore cakes (5 mm) were used to inoculate a 250 ml flask having medium volume of 40 ml and then incubated at 28 °C with agitation speed of 160 rpm for 48 h.

### Fermentation technique

For the production of antifungal compounds, 40 ml of fermentation medium [47 g soluble starch, 3 g yeast extract, 22 g peanut meal, 2.7 g (NH_4_)_2_ SO_4_, 2.7 g NaCl, 2.7 g CaCO_3_ dissolved in 1 L distilled water and pH was adjusted to 6.8–7.2] was taken in 250 ml flask and sterilized. After sterilization, the medium was inoculated with 5% (v/v) seed culture and incubated at 28 °C in rotatory shaker with agitation speed of 160 rpm for 96 h. After the termination of fermentation process, the culture was centrifuged and the supernatant was stored at −4 °C for further work (Gao et al. 2015).

### Antifungal activity

Antifungal activities were determined by oxford cup method (Wang et al. 2010a, b) and measured the inhibition zone.

### Stability test of the cultural filtrate of *Streptomyces* KX852460

Thermal stability, pH stability, illuminated light stability, and UV light stability were performed according to Zhao and Wu (2006). All the experiment were performed in triplicates and antifungal activity determined by oxford cup method mentioned above.

### Extraction of the culture filtrate

The culture filtrate (500 ml) was extracted two times with ethyl acetate as solvent. The solvent was added to the filtrate in the ratio of 1:1(v/v) and shaken vigorously for 20 min. The ethyl acetate phase that contains antibiotic was separated from the aqueous phase using separating funnel. Ethyl acetate layer was concentrated by evaporating to dryness at 50 °C and residue obtained was purified using methanol to (1.8 g) brown crude extract (Ahmed 2007).

### Purification and identification of the compound

The purification of the antimicrobial compound was carried out using silica gel column chromatography as described by Atta et al. (2009). Ethyl acetate was used as eluting solvent. The column was packed with silica gel (60–120 mesh). The sample to be separated was loaded on the packed column and eluted with the solvent at the flow rate of one drop per minute. A conical flask was placed at the bottom of the column to collect the eluted fractions. Antifungal activity was checked and most active fractions were used for further analysis. The antifungal compounds were identified by using gas chromatography–mass spectrometer technique (GC–MS). Agilent technologies 6890–5973 N with capillary column TG-5 ms Phenyl Methyl Siloxane (30 m × 250 μm × 0.25 μm) system were used. Mass detector used in split mode, and helium gas with flow rate of 1.0 ml/min was used as a carrier. Injector was operated at 230 °C and oven temperature for initial setup was 60 °C for 2 min, ramp 10/min to 280 °C for 8 min.

## Results

**Fig. 1:**
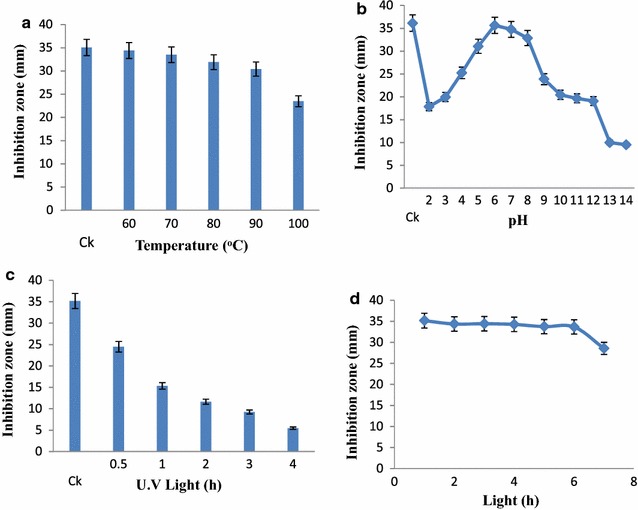
Effect of temperature (**a**), pH (**b**), illuminated light (**c**) and ultra violet light (**d**) on the stability of fermentation broth. *Ck* represents control of each treatment

**Fig. 2:**
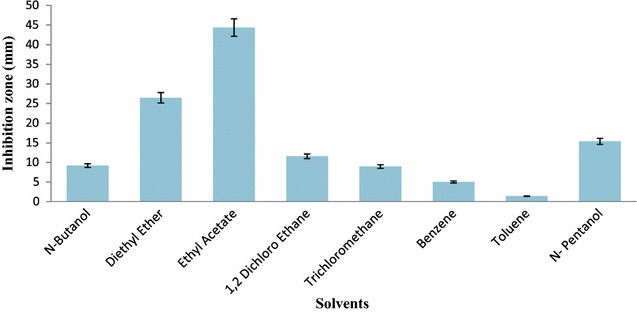
Effect of different solvents on activity of bioactive compound produced from *Streptomyces* strain KX852460

**Fig. 3:**
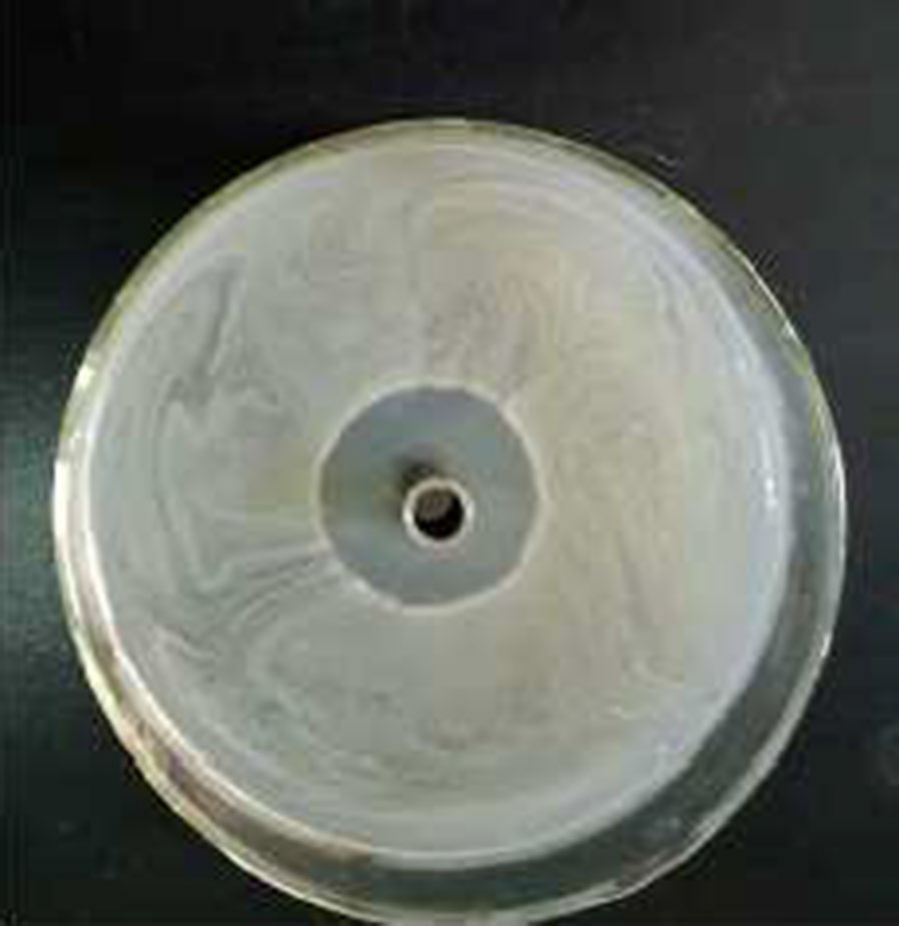
Activity of solvent extracted supernatant with ethyl acetate against *R. solani* AG-3

**Fig. 4:**
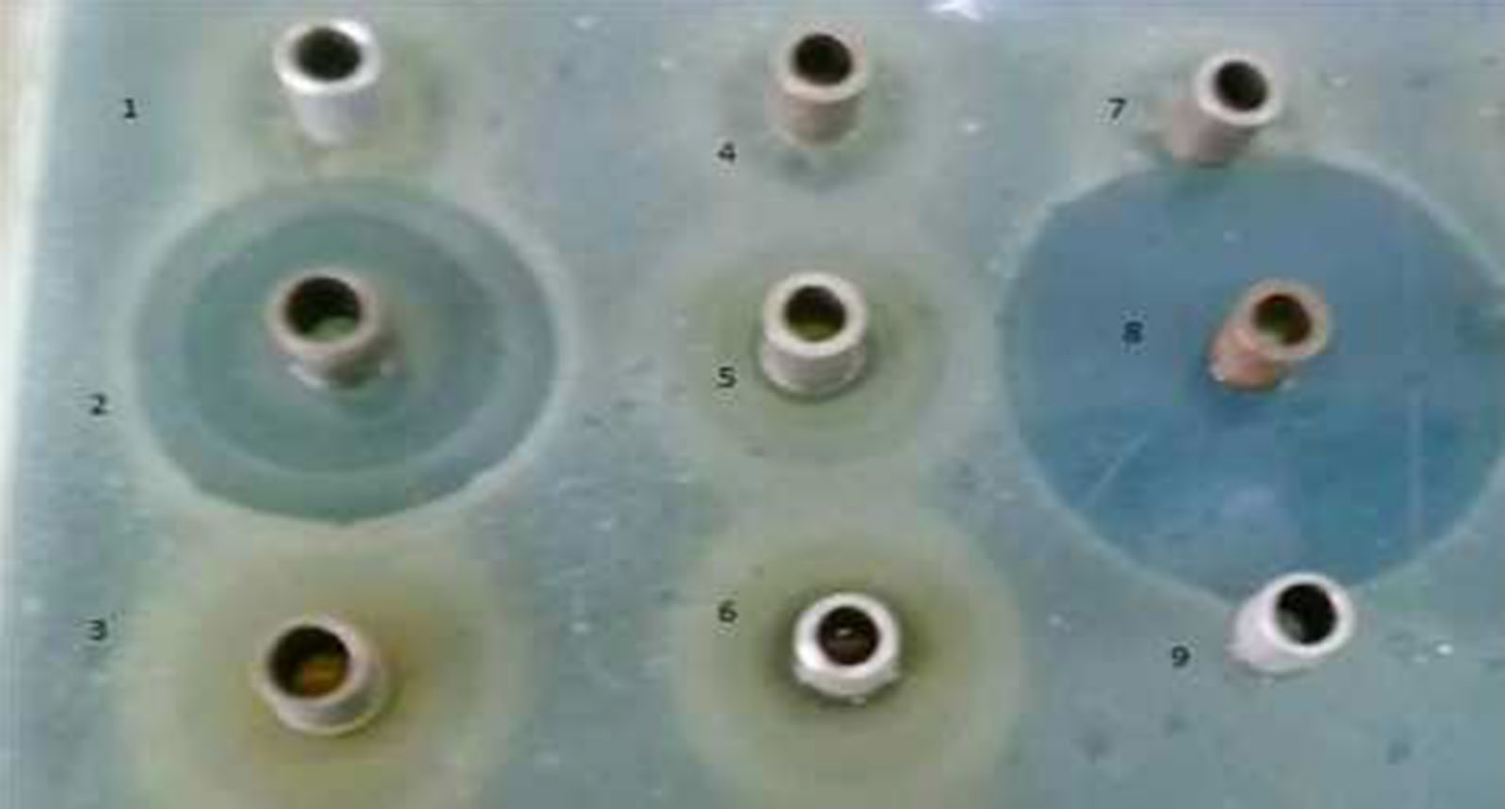
Antifungal activity of purified fractions by silica gel column chromatography and *1*, *2*, *3*, *4*, *5*, *6*, *7*, *8*, and *9* represented the numbers of purified fractions, obtained by silica gel column chromatography

**Fig. 5:**
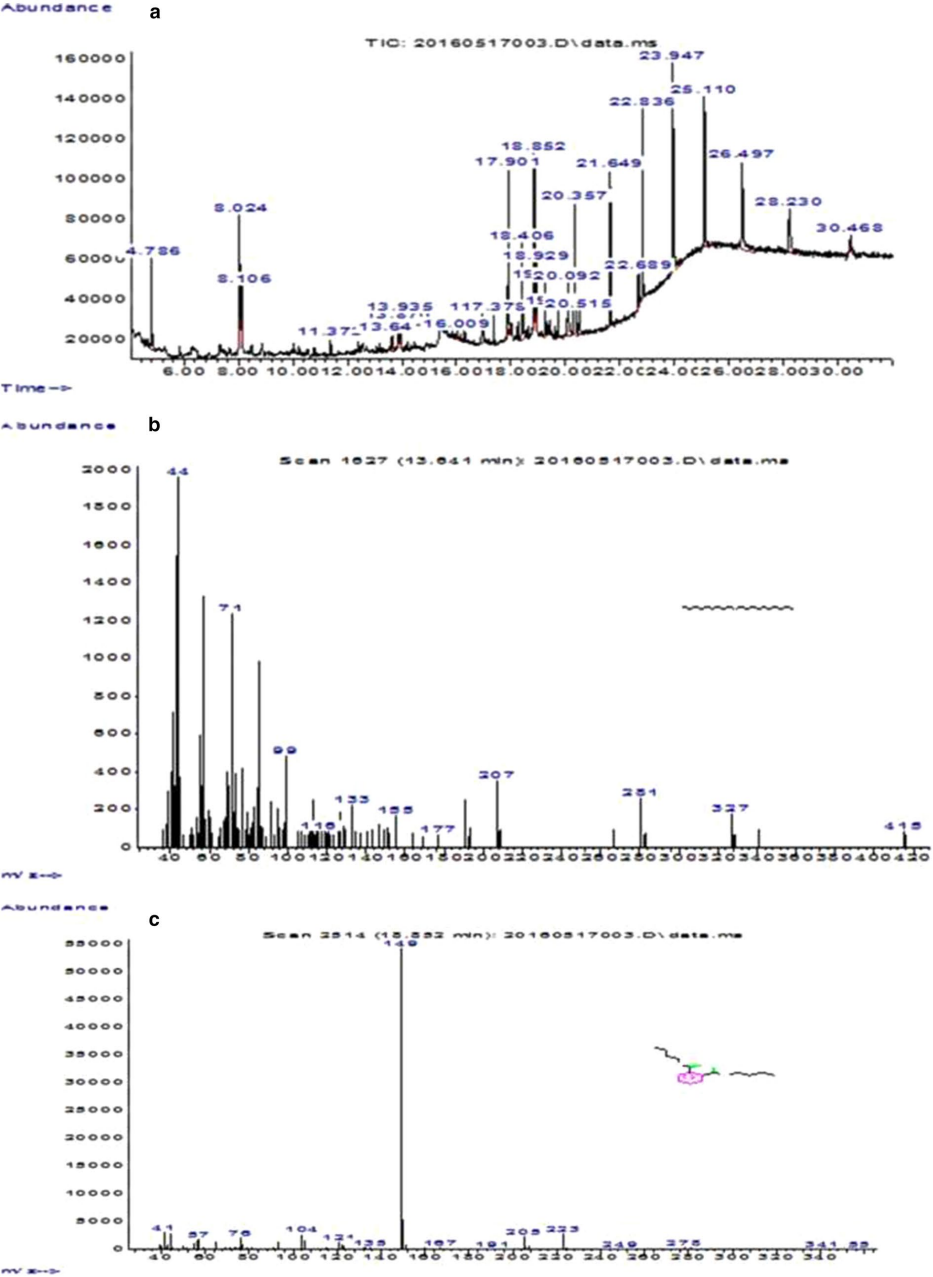
Gas chromatography-mass spectrometer (GC–MS) analysis of the purified active fraction (**a**), detection of eicosane (**b**) and dibutyl phthalate (**c**) from purified active fraction

*Streptomyces* strain KX852460 was isolated from the soil and screened against the *R. solani* AG-3 that is the causal agent of target spot in tobacco leaf. This strain had great potency against the pathogen. Twenty litter fermentation broth was produced by the *Streptomyces* strain KX852460 and broth was active against different plant pathogens including *R. solani* AG-3 having inhibition zone diameter of 45.78 mm. The strain also showed strong activity against *Sclerotinia sclerotiorum* with inhibition zone diameter of 50.4 mm (Table 1). Antifungal activity of the fermentation broth was found stable at various temperature levels from 60 to 90 °C, while at 100 °C activity was decreased (Fig. 1a). At different pH values antifungal activity of fermentation broth was observed, having peak activity at pH 6, while extreme pH conditions (pH 2, 14) resulted decreased antifungal activity (Fig. 1b). Fermentation broth treated with illuminated light showed stability in the activity against the pathogen (Fig. 1c). Under UV light treated broth was affected within duration of treatment and activity decreased abruptly (Fig. 1d). Solvent extraction method used to extract the bioactive compounds with several organic polar and non-polar solvents. All the extracts showed some inhibition effect against the pathogen ranging from 1.43 to 44.36 mm inhibition zone. But extract with ethyl acetate showed strong antifungal activity against the *R. solani* AG-3 KX852461 (Fig. 2). For further work based on this result ethyl acetate was selected and resulted antifungal activity against *R. solani* AG-3 KX852461 (Fig. 3). Crude extract dried at 50 °C and brown color powdery substance obtained that further purified by silica gel column chromatography by using ethyl acetate as eluent. Several fractions were obtained and most active fractions against the *R. solani*. In (Fig. 4) fraction number 8 had strongly inhibited the *R. solani* AG-3 with diameter of inhibition zone 52 mm. Active fraction was further analyzed by gas chromatography–mass spectrometer (GC–MS). GC–MS analysis detected 27 bioactive compounds (Table 2). By comparison of mass spectra of the constituent with NIST library twenty-seven peaks obtained (Fig. 5a). Among 27 different compounds, 16 compounds were the constituent of aromatic compounds while others were derivatives of different hydrocarbons. Eicosane (C_20_H_42_) and dibutyl phthalate (C_16_H_22_O_4_), having retention time of 13.641 (Fig. 5b) 18.852 (Fig. 5c) respectively were two antifungal compounds identified.

**Table 1:** Antimicrobial effects of the cultural filtrate

Test pathogens	Inhibition spectrum (mm) of cultural filtrate
*Alternaria alternata*	33.96
*Botrytis cinerea*	40.16
*Alternaria solani*	38.62
*Rhizoctonia solani* AG-3	45.78
*Fusarium oxysporum*	24.78
*Sclerotinia sclerotiorum*	50.4
*Bipolaris maydis*	33.72
*Colletotrichum capsici*	25.48

**Table 2:** Compounds identified in ethyl acetate extract of *Streptomyces* KX852460 by GC–MS

Peak #	Retention time	Area %	Name of the compound	Chemical formula	Molecular weight
1	4.786	3.92	Benzoic acid, 2-methoxy-, methyl ester	C_9_H_10_O_3_	166
2	8.024	5.36	Undecane	C_11_H_24_	156
3	8.106	1.8	Undecane	C_11_H_24_	268
4	11.373	0.98	Cyclohexasiloxane, dodecamethyl-	C_12_H_36_O_6_Si_6_	444
5	13.641	0.98	Eicosane	C_20_H_42_	282
6	13.87	0.72	Phenol, 2,4-bis(1,1-dimethylethyl)	C_14_H_22_O	206
7	13.935	1.02	Butylated hydroxytoluene	C_15_H_22_O	220
8	16.009	0.74	*N*-(Glycyl)alanine	C_5_H_10_N_2_O_3_	146
9	16.955	1.98	1,3-Diphenyl-4H-1,2,4-triazoline-5-thione	C_14_H_11_N_3_S	253
10	17.378	1.09	Cyclononasiloxane, octadecamethyl-	C_18_H_54_O_9_Si_9_	666
11	17.901	5.9	1,2-Benzenedicarboxylic acid, bis (2-methylpropyl) ester	C_16_H_22_O_4_	278
12	18.406	4.67	Hexadecanoic acid, methyl ester	C_17_H_34_O_2_	270
13	18.852	6.44	Dibutyl phthalate	C_16_H_22_O_4_	278
14	18.929	1.63	Cyclodecasiloxane, eicosamethyl-	C_20_H_66_O_10_Si_10_	740
15	19.264	2.04	p-Dicyclohexylbenzene	C_18_H_26_	242
16	19.74	1.1	p-Dicyclohexylbenzene	C_18_H_26_	242
17	20.092	3.44	9,10-Anthracenedione, 2-ethyl-	C_16_H_20_O_2_	236
18	20.357	5.95	Cyclodecasiloxane, eicosamethyl-	C_20_H_60_O_10_Si_10_	740
19	20.515	1.4	Octahydrotripheylene	C_18_H_20_	236
20	21.649	5.35	7-Chloro-10-ethyl-1-[[2-[[2-hydroxyethyl] amino] ethyl] amino]-3-[4-	C_26_H_25_ClF_3_N_3_O_2_	503
21	22.689	1.48	Hexanedioic acid, bis(2-ethylhexyl) ester	C_22_H_42_O_4_	370
22	22.836	6.56	Morphinan, 7,8-didehydro-4,5-epoxy-17-methyl-3, 6-bis [(trimethylsilyl) oxy]-, (5.alpha. 6. Alpha.)-	C_23_H_35_NO_3_Si_2_	429
23	23.947	14.46	Cyclononasiloxane, octadecamethyl-	C_18_H_54_O_9_Si_9_	666
24	25.11	7.55	Benzoic acid, 2,5-bis(trimethylsiloxy)-, trimethylsilyl ester	C_16_H_30_O_4_Si_3_	370
25	26.497	8.32	1,2,4-Benzenetricarboxylic acid, 4-butyl 1,2-dimethyl ester	C_15_H_18_O_6_	294
26	28.23	4.15	Cyclotrisiloxane, hexamethyl-	C_6_H_18_O_3_Si_3_	222
27	30.468	0.97	Cyclotrisiloxane, hexamethyl-	C_6_H_18_O_3_Si_3_	222

## Discussion

In this study *Streptomyces* strain KX852460 was screened against the *R. solani* AG-3 that is the causal agent of target spot disease in tobacco. Strain KX852460 belongs to *Streptomyces* which strongly inhibits the pathogen and could be curing the target spot in tobacco. The effects of the *R. solani* diseases are very severe throughout the world and affected the quality and yield of the several crops. For the control of *R. solani*, bacterial antagonist could be an environment friendly substituent. Various bacterial antagonists against the *R. solani*, including *Bacillus subtilis* CA32 in eggplant (Abeysinghe 2009), *Pseudomonas fluorescens* In5 (Michelsen and Stougaard 2011), *Burkholderia cepacia* T1A-2B and *Pseudomonas* sp. T4B-2A in tomato (De Curtis et al. 2010), inoculum of GB7 and 3Re4-18 in lettuce (Grosch et al. 2012), *endophytic Streptomyces* damping off growth promotion in tomato (Goudjal et al. 2014) have been reported as effective biological control agents. Antagonist activity of *Streptomyces* sp. CACIS-1.16CA against different phytopathogens including *R. solni* was also investigated by Evangelista-Martínez (2014).

In this study stability of the active cultural filtrate was determined at various temperatures, pH values, and treated with illuminated light and UV light. At 60–90 °C the antifungal activity of cultural filtrate of the *Streptomyces* strain KX852460 remained same, however above these conditions, the activity become decreased. pH values remained stable between 5.0 and 8.0 pH, while pH values above and lower beyond this were not stable. Uddin et al. (2013) reported stability of antimicrobial filtrate at different temperature and pH values. Treated with illumination light cultural filtrate was stable and showed good activity. Treated with UV light for long time was not stable, while treated for 30 min and for 1 h was stable. Stability of antimicrobial cultural filtrate from *Streptomyces* was also reported by Zhao and Wu (2006).

The crude extract obtained by solvent extraction with ethyl acetate showed strong activity against the *R. solani*. Isolation of crude extract by solvent extraction is very important phenomenon, to find a good solvent that have the potential to extract high yield and most potent bioactive compounds. Studies demonstrated that the extract of ethyl acetate have wide antimicrobial spectrum against the bacterial and fungus pathogens (Khamna et al. 2009; Kobayashi et al. 1994). Extract from *Streptomyces* EF37141 contains 27 different organic compounds. GC–MS analysis showed that the majority of the compounds were derivatives of the aromatic compounds. These compounds were antimicrobial and antifungal. Volatile organic compounds and polycyclic aromatic derivatives have the antifungal potential (Müller et al. 2009; Memić et al. 2011).

GC–MS is a novel technique to identify the secondary metabolites from the *Streptomyces* fermentation broth and analysis of GC–MS is very reliable to identify the compound in complex biochemical product. From current study some compounds were reported antifungal. Eicosane reported as antifungal compound (Karanja et al. 2012; Nandhini 2015) and dibutyl phthalate also reported antifungal compound (Nandhini 2015; Roy et al. 2006). Morphinan, 7,8-didehydro-4,5-epoxy-17-methyl-3,6-bis[(trimethylsilyl)oxy]-, (5.alpha, 6.alpha)-, cyclononasiloxane, octadecamethyl- and 1,2,4-benzenetricarboxylic acid, 4-butyl 1,2-dimethyl ester showed highest peaks and the area percent of these compounds also more than other compounds. On the base of GC–MS analysis highest peak number and area percent indicated that these three compounds were major in the extract of *Streptomyces* EF37141. These compounds consider active substances against the *R. solani*. Extract from the *Streptomyces* shows the same effectiveness as the oxine benzoate and fungicide (Sabaratnam and Traquair 2002).

## Abbreviations

PDA: potato dextrose agar
GC–MS: gas chromatography–mass spectrophotometry
NMR: nuclear magnetic resonance

## References

[CR1] Abeysinghe S (2009). Effect of combined use aí *Bacillus subtilis* CA32 and *Trichoderma harzianum* RUOI on biological control of *Rhizoctonia satani* on *Solanum melongena* and *Capsicum annuum*. Plant Pathol J.

[CR2] Ahmed AA (2007). Production of antimicrobial agent by *Streptomyces violachromogenes*. Saudi J Biol Sci..

[CR3] Atta H, Dabour S, Desoukey S (2009). *Sparsomycin* antibiotic production by *Streptomyces* sp. AZ-NIOFD1: taxonomy, fermentation, purification and biological activities. Agric Environ Sci.

[CR4] Awla HK, Kadir J, Othman R, Rashid TS, Wong MY (2016). Bioactive compounds produced by *Streptomyces* sp. isolate UPMRS4 and antifungal activity against *Pyricularia oryzae*. Am J Plant Sci.

[CR5] Bhavana M, Talluri VP, Kumar KS, Rajagopal S (2014). Optimization of culture conditions of *Streptomyces carpaticus* (MTCC-11062) for the production of antimicrobial compound. Int J Pharm Pharm Sci.

[CR6] Castano R, Borrero C, Trillas M, Avilés M (2013). Selection of biological control agents against tomato *Fusarium* wilt and evaluation in greenhouse conditions of two selected agents in three growing media. BioControl.

[CR7] De Curtis F, Lima G, Vitullo D, De Cicco V (2010). Biocontrol of *Rhizoctonia solani* and *Sclerotium rolfsii* on tomato by delivering antagonistic bacteria through a drip irrigation system. Crop Prot.

[CR8] Demain AL (2009). Antibiotics: natural products essential to human health. Med Res Rev.

[CR9] dos Reis Almeida FB, Cerqueira FM, Silva RN, Ulhoa CJ, Lima AL (2007). Mycoparasitism studies of *Trichoderma harzianum* strains against *Rhizoctonia solani*: evaluation of coiling and hydrolytic enzyme production. Biotechnol Lett.

[CR10] Evangelista-Martínez Z (2014). Isolation and characterization of soil *Streptomyces* species as potential biological control agents against fungal plant pathogens. World J Microbiol Biotechnol.

[CR11] Gao X, He Q, Jiang Y, Huang L (2015). Optimization of nutrient and fermentation parameters for antifungal activity by *Streptomyces lavendulae* Xjy and its biocontrol efficacies against *Fulvia fulva* and *Botryosphaeria dothidea*. J Phytopathol.

[CR12] Goudjal Y, Toumatia O, Yekkour A, Sabaou N, Mathieu F, Zitouni A (2014). Biocontrol of *Rhizoctonia solani* damping-off and promotion of tomato plant growth by endophytic actinomycetes isolated from native plants of Algerian Sahara. Microbiol Res.

[CR13] Grosch R, Dealtry S, Schreiter S, Berg G, Mendonça-Hagler L, Smalla K (2012). Biocontrol of *Rhizoctonia solani*: complex interaction of biocontrol strains, pathogen and indigenous microbial community in the rhizosphere of lettuce shown by molecular methods. Plant Soil.

[CR14] Jalaluldeen AM, Sijam K, Othman R, Ahmad ZAM (2015). Growth characteristics and production of secondary metabolites from selected *Streptomyces* species isolated from the Rhizosphere of Chili Plant. Int J Anh Res Sci Techol Eng.

[CR15] Johnk JS, Jones R, Shew H, Carling D (1993). Characterization of populations of *Rhizoctonia solani* AG-3 from potato and tobacco. Phytopathol.

[CR16] Kämpfer P, Dworkin M (2006). The family Streptomycetaceae, part I: taxonomy. The prokaryotes.

[CR17] Karanja E, Boga H, Muigai A, Wamunyokoli F, Kinyua J, Nonoh J (2012) Growth characteristics and production of secondary metabolites from selected novel *Streptomyces* species isolated from selected Kenyan national parks. In: Scientific conference proceeding

[CR18] Khamna S, Yokota A, Peberdy JF, Lumyong S (2009). Antifungal activity of *Streptomyces* spp. isolated from rhizosphere of Thai medicinal plants. Int J Integr Biol.

[CR19] Khattab AI, Babiker EH, Saeed HA (2016). *Streptomyces:* isolation, optimization of culture conditions and extraction of secondary metabolites. Int Curr Pharm J.

[CR20] Kobayashi A, Koguchi Y, Kanzaki H, Kajiyama S, Kawazu K (1994). A new type of antimicrobial phenolics produced by plant peroxidase and its possible role in the chemical defense systems against plant pathogens. Z Naturforsch C.

[CR21] Memić M, Selović A, Sulejmanović J (2011). Antifungal activity of polycyclic aromatic hydrocarbons against *ligninolytic* fungi. Hem Ind.

[CR22] Michelsen CF, Stougaard P (2011). A novel antifungal *Pseudomonas fluorescens* isolated from potato soils in Greenland. Curr Microbiol.

[CR23] Müller H, Westendorf C, Leitner E, Chernin L, Riedel K, Schmidt S, Eberl L, Berg G (2009). Quorum-sensing effects in the antagonistic rhizosphere bacterium *Serratia plymuthica* HRO-C48. FEMS Microbiol Ecol.

[CR24] Nandhini SU (2015). Gas chromatography–mass spectrometry analysis of bioactive constituents from the marine *Streptomyces*. Asi J Pharm Clin Res.

[CR25] Narasaiah BC, Leelavathi V, Sudhakar G, Mariyadasu P, Swapna G, Manne AK (2014). Isolation and structural confirmation of bioactive compounds produced by the strain *Streptomyces albus* CN-4. IOSR J Pharm Biol Sci.

[CR26] Roy R, Laskar S, Sen S (2006). Dibutyl phthalate, the bioactive compound produced by *Streptomyces albidoflavus* 321.2. Microbiol Res.

[CR27] Sabaratnam S, Traquair JA (2002). Formulation of a *Streptomyces* biocontrol agent for the suppression of *Rhizoctonia* damping-off in tomato transplants. Biol Control.

[CR28] Snyder LR, Kirkland JJ, Glajch JL (2012). Practical HPLC method development.

[CR29] Tiwari V, Roy R, Tiwari M (2015). Antimicrobial active herbal compounds against *Acinetobacter baumannii* and other pathogens. Front Microbiol.

[CR30] Uddin M, Mahmud N, Anwar N, Manchur MA (2013). Bioactive metabolite production by *Streptomyces albolongus* in favourable environment. J Microbiol Infect Dis.

[CR31] Wang X, Huang L, Kang Z, Buchenauer H, Gao X (2010). Optimization of the fermentation process of *actinomycete* strain Hhs. 015 T. Biomed Res.

[CR32] Wang YH, Fang XL, Li YP, Zhang X (2010). Effects of constant and shifting dissolved oxygen concentration on the growth and antibiotic activity of *Xenorhabdus nematophila*. Biores Technol.

[CR33] Wu YH, Zhao YQ, Fu Y, Zhao XX, Chen JG (2012). First report of target spot of flue-cured tobacco caused by *Rhizoctonia solani* AG-3 in China. Plant Dis.

[CR34] Xue L, Xue Q, Chen Q, Lin C, Shen G, Zhao J (2013). Isolation and evaluation of rhizosphere *actinomycetes* with potential application for biocontrol of *Verticillium* wilt of cotton. Crop Prot.

[CR35] Zeng Q, Huang H, Zhu J, Fang Z, Sun Q, Bao S (2013). A new nematicidal compound produced by *Streptomyces albogriseolus* HA10002. Antonie Van Leeuwenhoek.

[CR36] Zhao G, Wu YH (2006). Antimicrobial spectrum and stability of the fermentation broth of *Streptomyces*. J Agrochem.

